# Resonance frequency analysis of dental implants placed at the posterior maxilla varying the surface treatment only: A randomized clinical trial

**DOI:** 10.1111/cid.12510

**Published:** 2017-06-20

**Authors:** Marcelo M. Novellino, Newton Sesma, Piero R. Zanardi, Dalva C. Laganá

**Affiliations:** ^1^ Department of Prosthodontics, School of Dentistry University of São Paulo São Paulo Brazil

**Keywords:** clinical research, implant stability, implant surface, randomized controlled trial, surface properties

## Abstract

**Background:**

Chemical modifications of the dental implant surface that improve the wettability result in a faster and better osseointegration.

**Purpose:**

The aim of this randomized clinical trial was to evaluate the implant stability quotient (ISQ) of implants with similar designs, treated with 2 surfaces, sandblasted acid‐etched (SAE) and hydrophilic SAE, within the initial 16 weeks of healing.

**Material and methods:**

A total of 64 implants (32 SAE—control group and 32 modified SAE—test group) with the same design, length, and diameter (conical and compressive, 4.3 × 10 mm) were inserted into the posterior maxillae of 21 patients partially edentulous. The ISQ values were collected at post‐surgery (T0), 1 week (T1), 2 weeks (T2), 3 weeks (T3), 5 weeks (T4), 8 weeks (T5), 12 weeks (T6), and 16 weeks (T7).

**Results:**

None of the implants failed. Test group presented ISQ values higher than the control group (ANOVA—*P* < .01) from T5 to T7. When comparing groups regarding the amount of time required to achieve ISQ ≥ 70 as a reference, there was a statistically significant difference (cox regression—*P* < .01), and a hazard ratio of 2.24 (CI 1.62‐3.11). At the 1‐year follow‐up, there was a drop out of 1 patient, and 2 implants were no longer evaluated. Survival rate for both groups was 100% at the 1‐year follow‐up.

**Conclusions:**

The current study suggests that implants with hydrophilic surface (modified SAE) integrate faster than implants with SAE surface. The stability gain of the test group was 2.24 times faster than the control group after 5 weeks of evaluation at the posterior region of the edentulous maxillae.

## 1 INTRODUCTION

Studies have proved that implants with treated surfaces, such as sandblasted and acid‐etched (SAE), result in faster osseointegration compared to implants with machined surfaces.^1^, ^2^ Also, chemical modifications used to improve the wettability of such surfaces result in faster osseointegration.^1^, ^3^ The wettability of a clean hydrophilic titanium oxide surface is obtained by an extensive hydroxylation/hydration of the oxide layer, and this leads to an interaction between the titanium surface and water, which allows biomolecules, such as proteins, to be adsorbed.^2^, ^4^ The wetting properties of the implant surface can be examined experimentally by contact angles, and it can be determined by the surface chemical composition and by roughness.^4^ Chemical modifications of the titanium surface also have an influence on the surface charge, which may affect the protein adsorption, cell adhesion, and specific cell responses.^5^

SAE and chemistry‐modified SAE implants surface have shown the same morphologic microstructure and a similar roughness surface,^6^, ^7^, ^8^ although SAE implant surface has a major amount of carbon and less oxygen than chemistry‐modified SAE surface.^6^, ^8^

Dental implant stability is defined as the absence of clinical mobility, and this can also be suggested as a definition of osseointegration.^9^ Resonance frequency analysis (RFA), which was developed by Meredith and colleagues, provides a clinical, noninvasive, and nondestructive method to assess the implant stability and osseointegration.^10^, ^11^

The aim of this randomized clinical trial was to statistically compare the implant stability quotient (ISQ) results obtained by implants of the same design, length, and diameter with SAE surface and SAE chemically modified (hydrophilic) surface placed at the posterior area of the maxilla within the initial 16 weeks of follow‐up.

## 2 MATERIALS AND METHODS

The study protocol was submitted and approved by the Ethical Committee of São Paulo University School of Dentistry, Brazil (CAAE 18911913.7.0000.0075). The research was also registered at the Clinical Trials web site (http://www.clinicaltrials.gov) with the identification number NCT02134743 and the name *Evaluation of the stability of implants with two different surface treatments*. A total of 21 healthy nonsmoking patients (aged 27–64 years, 8 male and 13 female, mean age = 49 ± 3.6 years), with no systemic contraindications (ASA I) to implant placement, and with one or more edentulous areas in the posterior maxilla (premolar and molar), a subantral bone height of ≥8 mm, a width of the residual ridge ≥6.3 mm, bone density of D3 or D4 (as classified by Lekholm and Zarb^12^) and at least a 3‐month post‐extraction healing period were included in the study. The exclusion criteria were as follows: previous bone grafting and/or sinus lift, uncontrolled diabetes, untreated periodontitis, severe bruxing or clenching habits, pregnancy/breastfeeding, recent bisphosphonates use, alcohol or drug addiction, and history of local radiation therapy. The study was carried out from December 2013 to July 2015 at the Prosthesis and Implant Clinic of São Paulo University Dental School.

A total of 64 implants, 32 with SAE surface (Drive CM Neoporos, Neodent, Curitiba, Brazil) and 32 with a modified SAE surface (Drive CM Acqua, Neodent) were placed and evaluated by Osstell (Integration Diagnostic AB, Goteborg, Sweden) within a period of 16 weeks (T0: immediate postsurgical, T1: 1 week postsurgical, T2: 2 weeks, T3: 3 weeks, T4: 5 weeks, T5: 8 weeks, T6: 12 weeks, and finally T7: 16 weeks).

### 2.1 Clinical and surgical procedures

The preoperative examination included a panoramic radiograph, clinical examination, models for diagnosis, a radiograph template with metal marks, and a cone beam computer tomography with software for planning (Dental Slice, Bioparts, Brasília, Brazil). All surgical procedures were carried out under local anesthesia by the same surgeon (MMN). Preoperative antibiotic prophylaxis was provided 1 day before the procedure and for 7 days (Amoxicillin 500 mg). All implants were placed according to the manufacturer's recommendation and after a midcrestal incision and mucoperiostal flap elevation of both faces (buccal and lingual). Implants of the same diameter and length, 4.3 mm and 10 mm, respectively, were placed in a one‐stage procedure; special developed healing screws (Neodent), with a connection for a Smarpeg type A3 (Integration Diagnostic AB), were installed on top. The special healing screws remained in place for 16 weeks, while avoiding micro‐movements or early torque on implants during the time of the data collection. No prosthetic procedure was performed before 16 weeks.

### 2.2 Resonance frequency measurements and clinical assessment

**Figure 1:**
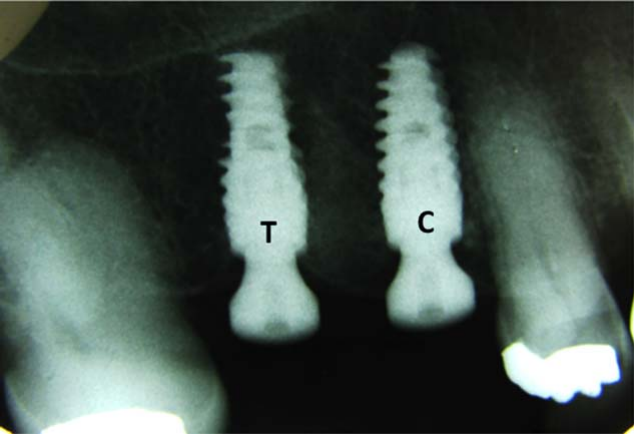
Implants with the same characteristics but with different surfaces in the same patient. T for the test group and C for the control group

Buccal and mesial measurements were performed after placing the smartpegs on the healing screws. All arithmetic mean obtained by the 2 ISQ values were registered for the respective time. All implants were followed for 16 weeks with Osstell. All implants were clinically followed, with periapical x‐rays and peri‐implant measurements taken for 1 year after the surgery of implant placement to follow the implant survival rate (Figure 1).

### 2.3 Randomization and implant allocation

In this study, patients were not allocated to groups, but the implant sites were divided into 2 groups: the test group (an Acqua surface) and the control group (a Neoporos surface). Randomization was performed through the web site Research Randomizer (https://www.randomizer.org) using a single block of 64 numbers, a procedure known as simple randomization.^13^ All sites were included according to the sequence of the patients' appointments in correspondence with the numbers 1–64 of the single block randomization. If a patient had more than one edentulous area, the sequence that followed corresponded to the randomization block from quadrant 1 to 2, following the numerical order of the dental elements (14–27). The randomization provided numerical sequences for each block (1–64), with even numbers determining the placement of implants from the control group, and odd numbers determining the placement of implants from the test group.

Only the surgeon (MMN) and his assistant knew the distribution of the implants, and both the patients and the dentist (PRZ) who collected the data were blinded to the implant distribution.

### 2.4 Statistical analysis

Statistical analysis (ANOVA for repeated measures) was performed to compare groups, and for the interaction factor, the Greenhouse‐Geisser test was performed. A Kaplan‐Meier graphic was used to present the survival rate according to the time. For this test, an ISQ cut‐off was determined with the aim of comparing the ISQ variation of both curves and not as a determinant factor for osseointegration. The test was carried out to evaluate the surface treatment performance and not to evaluate implant loss or failure. The survival test results show how many implants achieve an ISQ ≥ 70 for each segmented time. Cox regression was also performed to evaluate the time‐dependency for both groups to achieve ISQ ≥ 70. In the same way, the hazard ratio was used to compare the “risk” of the dental implants achieving an ISQ ≥ 70.

## 3 RESULTS

### 3.1 Population results

Twenty‐one patients (8 men, 38.095% and 13 women, 61.905%) were assessed. The mean age was 49 ± 3.6 years. The youngest was 27 years old, and the oldest was 64 years old. Surgery and healing proceeded without complications, with a low level of postoperative discomfort for the whole group. During the evaluation of RFA (16 weeks), no patient dropouts were registered.

The average torque, rated at the time of implant installation, was 35.125 ± 4.498. The average torque for the test group was 34.656 ± 6.940, and for the control group, it was 36.063 ± 5.995.

### 3.2 Implant stability

**Figure 2:**
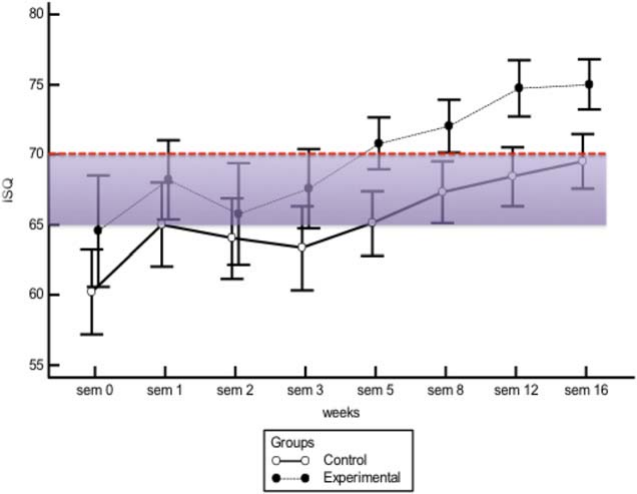
Mean ISQ values and confidence interval of control and test group week by week

The minimum and maximum RFA values during the study were 42 and 81 for the control group, and 32.5 and 82.5 for the test group, respectively. Figure 2 illustrates the mean and confidence interval of each group from week to week. The test group showed higher values than the control group from week 5 to 16 (Figure 2).

The test group and the control group's ISQ measurements resulted in statistically significant differences (ANOVA, repeated measurements). The interaction factor (weeks) was also statistically significant for both groups (*P* < .01). There was no statistically significant difference for the Greenhouse‐Geisser analysis (*P* = .150), and comparing the groups in terms of the interaction factor showed that the 2 curves behaved similarly (increase of ISQ according to time).

**Figure 3:**
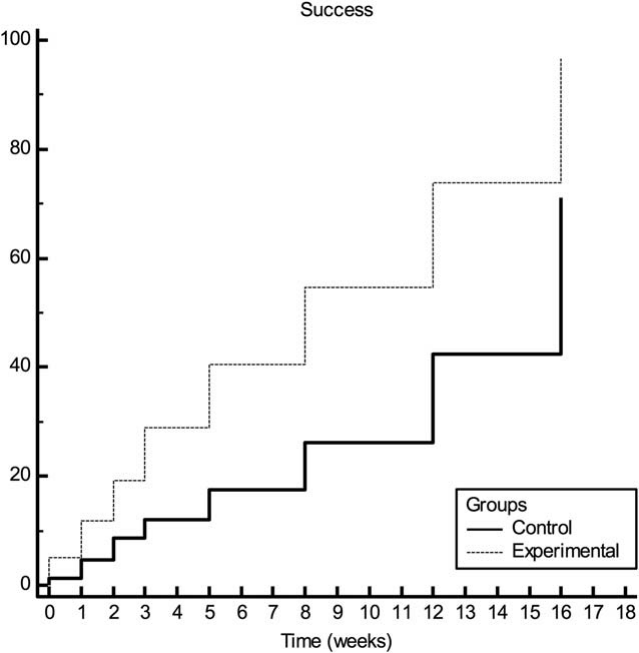
Kaplan‐Meier plot for test and control group, with the outcome being successful implant stability (ISQ ≥ 70)

Seventy was considered the ISQ reference for implant success, and Figure 3 presents the Kaplan‐Meier plot. A comparison of both groups using Cox regression and with regard to the time required for the implants to achieve ISQ ≥ 70 resulted in a statistically significant difference (*P* < .01), and a hazard ratio (hour) of 2.24 (CI 1.62–3.11). Regarding the amount of time an implant took to reach ISQ ≥ 70, the test group had a statistically significant difference compared to the control group, and the test group was 2.24 times faster than the control group.

### 3.3 One year follow‐up

Of the 21 patients, 1 patient with 2 implants did not show up for the 1‐year follow‐up after the implant placement. At the time of the evaluation, 29 implants (46.77%) had not yet been restored; none had mobility or pain when a torque was applied to the abutments; neither was there mobility or pain with the use of percussion (on the restored implants). Seven implants (11.29%) had bleeding on probing, but none had suppuration. Only 1 implant (1.61%) had significant bone loss according to the radiographs possibly because a poorly adapted restoration.

## 4 DISCUSSION

Although it was not a split‐mouth study, implants were randomized and allocated to respective groups. This resulted in a situation in which different implants (C and T) were placed for the same patients, with their own biological characteristics, general health conditions, and habits.

According to Guller and colleagues, gender can interfere with ISQ values, with men usually having higher values.^10^ Conversely, Ostman and colleagues point out that the differences in the RFA values between genders are not clinically significant. Furthermore, there were not any differences in the failure rate between men and women.^14^ Other factors could influence implant stability; diameter, length, design, and region may affect the ISQ values. Sim and Lang showed that the implant length influenced the ISQ values, but the jaw region (bone quality) had greater importance for RFA analysis.^15^ There is no consensus about the implant diameter affecting the implant's stability; conversely, the design and region where the implants were placed seemed to affect the ISQ values.^14^, ^15^, ^16^ For this study, only implants with the same length, diameter, and design were installed. The region of the implant placement was always the same (posterior maxilla), although patients may have had differences in bone quality.

Clinically, RFA technology can be useful for monitoring the implant stability evolution throughout the healing process.^17^ An increase in ISQ values over time may reflect bone apposition and remodeling at the implant‐bone interface.^16^, ^18^ The maximum mean ISQ value of this study (82.5) is in agreement with other clinical studies, but the minimum is not (32.5). Han and colleagues obtained 84 as the highest ISQ value and 55 as the lowest ISQ value.^16^ Ersanli and colleagues found 82 and 57 as the highest and lowest ISQ values, respectively.^19^ The differences in the minimum ISQ values for the present study may be due to the authors having placed implants at the mandible and maxillae or to different lengths, diameters, and designs. Sim and Lang showed lower ISQ values for bone type III and IV than for types I and II.^12^, ^15^ In the present study, all implants were of the same design (Drive CM, 4.3 × 11), and they were placed at the posterior region of the upper maxillae.

An ISQ decrease was observed for both groups in the second and third weeks after the implant placement. The mean ISQ value started to increase progressively week by week after this initial period. These results agree with other studies^10^, ^16^, ^19^ and suggest the existence of a gap between the primary and secondary stability when the implant stability increases. This decrease in ISQ demonstrates the primary stability loss, and the recovery means that the secondary (or biological) stability has been established. Osseointegration occurs only after bone reabsorption processes, which lead to a decrease in mechanical stability for a short period of time^20^ and a subsequent decrease in ISQ values. The test group had the lowest value in the second week, while the control group had the lowest value in the third week. This was probably due to the chemistry modification of the implants' surfaces in the test group accelerating the biological osseointegration events. Van Eekeren and colleagues found similar results for implants with hydrophilic surfaces; as in this study, the dip in stability was highest in week 2.^21^ As in the case of this study, other authors have stated that implants without hydrophilic surfaces experience the lowest point of stability between the third and fourth week.^22^, ^23^ To minimize bias, all implants' design, length and diameter were standardized in the present study; furthermore, all fixtures were placed in the posterior area of the maxilla because the aim was to evaluate the clinical significance of the implants with different surfaces according to time.

In this research, the mean ISQ of the test group was found to be significantly different from the control group between the fifth and sixteenth week, as presented in Figure 2. It can be argued that implants with hydrophilic SAE surfaces showed faster and greater stability gain than implants with SAE surfaces. The gain in stability after the properly healing period can be considered as resulting from osseointegration itself, as described in the literature.^15^, ^16^ Sim and Lang speculated that the significant increase in ISQ values could represent the establishment of a biological adhesion, which would replace the mechanical primary stability.^15^

For both groups, the ISQ values rose steadily, as shown in the group comparison versus interaction factor, which was not statistically significant (Greenhouse‐Geisser); in other words, the mean ISQ values increased in time for both groups. It can be speculated that at the end of the healing process, both implant surfaces would show similar ISQ results. Han and colleagues demonstrated that implants with hydrophilic SAE surfaces from a different supplier than the one mentioned in this study presented ISQ values that were higher than implants with SAE surfaces produced by the same company mentioned at the beginning and in the middle of this study. However, at the end of the evaluations, there was no significant difference between the 2 surfaces. Therefore, hydrophilic surfaces could be more suitable for early loading.^16^ Lang and colleagues studied the bone‐to‐implant contact (BIC) of implants with SAE and wet SAE surfaces in humans over 7‐ to 42‐day periods. Implants with modified SAE had superior BIC after 14 and 28 days, but at the end of the 42‐day period, BIC values were equal and satisfactory for both types of surfaces.^3^

A faster and greater gain in stability is important for early loading protocols, as the gain in stability would represent greater and faster osseointegration. Sartoretto and colleagues evaluated the BIC and bone area fraction occupied (BAFO) levels of implants with hydrophilic and regular dry surfaces installed in the tibias of rabbits. Implants with modified surface (wettability) showed an increase in BIC and BAFO within the initial 14 days compared to implants without modification at 28 days.^6^ It was also found that the hydrophilic implants had 2‐times‐faster osseointegration. Clinical studies also confirm the possibility of using early loading protocols for implants with surface modified SAE. Bornstein and colleagues demonstrated in a retrospective clinical study that implants with modified SLA surfaces can be loaded in 3 weeks instead of 6 or 8.^24^ Roccuzzo and colleagues stated that successful functional loading of chemically modified titanium implants is possible at 3 weeks in the maxillary molar region.^25^ Also, it was seen in the past, when machined implants were installed more frequently, that implants placed in the posterior maxilla had higher failures rates compared to implants placed in others areas of the mouth; in particular, implants with treated surfaces had better success rates in the long term than implants with smooth surfaces.^26^ In this study, all implants (with an identical design) with treated surfaces placed in the posterior maxilla resulted in a 100% success rate. Therefore, active surface treatment could be an important way to enhance the success rate in areas of poor bone density.

In this study, it is not possible to state that the modified SAE‐surface implants had a higher or better osseointegration; it is only possible to say that it occurred more quickly. The fastest stability gain observed in the tested implants may represent an increase of the percent of BIC. Park and colleagues showed a significant correlation between ISQ values measured by RFA and the percent of BIC in the rabbit tibia model after 4 weeks of healing.^27^ However, Manresa and colleagues found a lack of correlation between ISQ values (as determined by RFA) and BIC (histomorphometrical data) in a study performed on dogs.^28^ Several factors determine the quality of the connection between bone and implant material, such as percent of BIC, bone density and implant length. However, these parameters could only be studied by means of histological analyses, which are an invasive technique.^21^

For this study, ISQ values ≥70 were defined as a parameter of implant success. This is because ≥70 can be considered an outcome that is higher than the minimum implant stability suggested by the manufacturers as suitable for immediate loading.^2^, ^29^, ^30^, ^31^ Also, Sennerby and Meredith considered ISQ values of between 55 and 65 at any time during the lifetime of the implant as an indication of a safe level of implant stability.^32^ The ≥70 ISQ value is only 1 parameter, and it is a cut‐off value for the statistical tests. The most important factor is that the stability increases with time, faster for the test group than for the control group and with a statistically significant difference, as can be seen in Figure 2.

Figure 3 shows the total number of implants with ISQ ≥ 70 within the 16 weeks. This number was higher for the test group than for the control at the end of the 16 weeks: almost 100% of the test group and just over 70% of the control group reached the defined parameter. Statistical analysis confirmed that the test group reached ISQ ≥ 70 at a speed 2.24 times faster than the control group. This is in agreement with other studies, which conducted histomorphometric, clinical, and RFA evaluations.^3^, ^6^, ^24^, ^25^

For the 1‐year follow‐up, the results were in agreement with other studies that showed SAE and modified‐SAE implants with a more than 95% survival rate.^24^, ^25^, ^33^ However, in this follow‐up, all implants were placed in the posterior area of the maxillae. Bone loss in the case of 1 implant, detected by x‐ray, was due to a poorly adapted restoration, so it did not relate to the implant itself.

Although the subject is currently being studied a great deal, more randomized clinical trials (RCT) are required to scientifically confirm the results described in the literature. Roccuzzo and colleagues said that more RCTs are required to verify the hypothesis that the modified SAE surface accelerates osseointegration and reduces failures in the initial healing phase.^25^ Chambrone and colleagues, in a systematic review, concluded that few RCTs on implants with hydrophilic surfaces are available for analysis. The authors concluded that there is insufficient evidence to support or refute significant differences between implants with the surfaces described.^34^

## 5 CONCLUSION

In conclusion, the results of the current study suggest that implants with hydrophilic surfaces osseointegrate faster than implants with SAE surfaces. The stability gain of implants with hydrophilic surfaces was 2.24 times faster than the group with the SAE surfaces, and an increased number of the tested implants had ISQ results ≥70 at 16 weeks. It is also possible to conclude that the Ostell device is an effective method to evaluate the osteointegration of implants during the healing process, and new research studies on modified SAE‐implant surfaces, especially RCTs, are necessary.

## References

[1] Alfarsi MA , Hamlet SM , Ivanovski S. Titanium surface hydrophilicity enhances platelet activation. Dent Mater J. 2014;33(6):749–756. 25311339 10.4012/dmj.2013-221

[2] Bornstein MM , Hart CN , Halbritter SA , Morton D , Buser D . Early loading of nonsubmerged titanium implants with a chemically modified sand‐blasted and acid‐etched surface: 6‐month results of a prospective case series study in the posterior mandible focusing on peri‐implant crestal bone changes and implant stability quotient (ISQ) values. Clin Implant Dent Relat Res. 2009;11(4):338–347. 19438966 10.1111/j.1708-8208.2009.00148.x

[3] Lang NP , Salvi GE , Huynh‐Ba G , Ivanovski S , Donos N , Bosshardt DD . Early osseointegration to hydrophilic and hydrophobic implant surfaces in humans. Clin Oral Implants Res. 2011;22(4):349–356. 21561476 10.1111/j.1600-0501.2011.02172.x

[4] Rupp F , Scheideler L , Eichler M , Geis‐Gerstorfer J . Wetting behavior of dental implants. Int J Oral Maxillofac Implants. 2011;26(6):1256–1266. 22167431

[5] Bosshardt DD , Salvi GE , Huynh‐Ba G , Ivanovski S , Donos N , Lang NP . The role of bone debris in early healing adjacent to hydrophilic and hydrophobic implant surfaces in man. Clin Oral Implants Res. 2011;22(4):357–364. 21561477 10.1111/j.1600-0501.2010.02107.x

[6] Sartoretto SC , Alves AT , Resende RF , Calasans‐Maia J , Granjeiro JM , Calasans‐Maia MD . Early osseointegration driven by the surface chemistry and wettability of dental implants. J Appl Oral Sci. 2015;23(3):279–287. 26221922 10.1590/1678-775720140483 PMC4510662

[7] Schwarz F , Wieland M , Schwartz Z , et al. Potential of chemically modified hydrophilic surface characteristics to support tissue integration of titanium dental implants. J Biomed Mater Res B Appl Biomater. 2009;88(2):544–557. 18837448 10.1002/jbm.b.31233

[8] Zinelis S , Silikas N , Thomas A , Syres K , Eliades G . Surface characterization of SLActive dental implants. Eur J Esthet Dent. 2012;7(1):72–92. 22319766

[9] Sennerby L , Roos J. Surgical determinants of clinical success of osseointegrated oral implants: a review of the literature. Int J Prosthodont. 1998;11(5):408–420. 9922733

[10] Guler AU , Sumer M , Duran I , Sandikci EO , Telcioglu NT . Resonance frequency analysis of 208 Straumann dental implants during the healing period. J Oral Implantol. 2013;39(2):161–167. 22103915 10.1563/AAID-JOI-D-11-00060

[11] Meredith N , Alleyne D , Cawley P. Quantitative determination of the stability of the implant‐tissue interface using resonance frequency analysis. Clin Oral Implants Res. 1996;7(3):261–267. 9151590 10.1034/j.1600-0501.1996.070308.x

[12] Lekholm UZ , Zarb GA. Patient selection and preparation In: Branemark P‐I, Zarb GA, Albrektsson T, eds. Tissue Integrated Prosthesis. Osseointegration in Clinical Dentistry. Chicago, IL: Quintessence Publishing Company; 1985:199–210.

[13] Moher D , Hopewell S , Schulz KF , et al. CONSORT 2010 explanation and elaboration: updated guidelines for reporting parallel group randomised trials. Int J Surg. 2012;10(1):28–55. 22036893 10.1016/j.ijsu.2011.10.001

[14] Ostman PO , Hellman M , Wendelhag I , Sennerby L . Resonance frequency analysis measurements of implants at placement surgery. Int J Prosthodont. 2006;19(1):77–83. 16479765

[15] Sim CP , Lang NP. Factors influencing resonance frequency analysis assessed by Osstell mentor during implant tissue integration: I. Instrument positioning, bone structure, implant length. Clin Oral Implants Res. 2010;21(6):598–604. 20666786 10.1111/j.1600-0501.2009.01878.x

[16] Han J , Lulic M , Lang NP. Factors influencing resonance frequency analysis assessed by Osstell mentor during implant tissue integration: II. Implant surface modifications and implant diameter. Clin Oral Implants Res. 2010;21(6):605–611. 20666787 10.1111/j.1600-0501.2009.01909.x

[17] Atieh MA , Alsabeeha NH , Payne AG. Can resonance frequency analysis predict failure risk of immediately loaded implants?. Int J Prosthodont. 2012;25(4):326–339. 22720282

[18] Degidi M , Perrotti V , Piattelli A , Iezzi G . Mineralized bone‐implant contact and implant stability quotient in 16 human implants retrieved after early healing periods: a histologic and histomorphometric evaluation. Int J Oral Maxillofac Implants. 2010;25(1):45–48. 20209186

[19] Ersanli S , Karabuda C , Beck F , Leblebicioglu B . Resonance frequency analysis of one‐stage dental implant stability during the osseointegration period. J Periodontol. 2005;76(7):1066–1071. 16018748 10.1902/jop.2005.76.7.1066

[20] Abrahamsson I , Berglundh T , Linder E , Lang NP , Lindhe J . Early bone formation adjacent to rough and turned endosseous implant surfaces. An experimental study in the dog. Clin Oral Implants Res. 2004;15(4):381–392. 15248872 10.1111/j.1600-0501.2004.01082.x

[21] van Eekeren P , Said C , Tahmaseb A , Wismeijer D . Resonance frequency analysis of thermal acid‐etched, hydrophilic implants during first 3 months of healing and osseointegration in an early‐loading protocol. Int J Oral Maxillofac Implants. 2015;30(4):843–850. 26252037 10.11607/jomi.3985

[22] Simunek A , Kopecka D , Brazda T , Strnad I , Capek L , Slezak R . Development of implant stability during early healing of immediately loaded implants. Int J Oral Maxillofac Implants. 2012;27(3):619–627. 22616056

[23] Makary C , Rebaudi A , Sammartino G , Naaman N . Implant primary stability determined by resonance frequency analysis: correlation with insertion torque, histologic bone volume, and torsional stability at 6 weeks. Implant Dent. 2012;21(6):474–480. 23149504 10.1097/ID.0b013e31826918f1

[24] Bornstein MM , Wittneben JG , Bragger U , Buser D . Early loading at 21 days of non‐submerged titanium implants with a chemically modified sandblasted and acid‐etched surface: 3‐year results of a prospective study in the posterior mandible. J Periodontol. 2010;81(6):809–818. 20450357 10.1902/jop.2010.090727

[25] Roccuzzo M , Wilson TG Jr. A prospective study of 3 weeks' loading of chemically modified titanium implants in the maxillary molar region: 1‐year results. Int J Oral Maxillofac Implants. 2009;24(1):65–72. 19344027

[26] Balshe AA , Assad DA , Eckert SE , Koka S , Weaver AL . A retrospective study of the survival of smooth‐ and rough‐surface dental implants. Int J Oral Maxillofac Implants. 2009;24(6):1113–1118. 20162117

[27] Park KJ , Kwon JY , Kim SK , et al. The relationship between implant stability quotient values and implant insertion variables: a clinical study. J Oral Rehabil. 2012;39(2):151–159. 21923718 10.1111/j.1365-2842.2011.02255.x

[28] Manresa C , Bosch M , Echeverría JJ. The comparison between implant stability quotient and bone‐implant contact revisited: an experiment in Beagle dog. Clin Oral Implants Res. 2014;25(11):1213–1221. 24102812 10.1111/clr.12256

[29] Kokovic V , Jung R , Feloutzis A , Todorovic VS , Jurisic M , Hammerle CH . Immediate vs. early loading of SLA implants in the posterior mandible: 5‐year results of randomized controlled clinical trial. Clin Oral Implants Res. 2014;25(2):e114–e119. 23278375 10.1111/clr.12072

[30] Ostman PO , Hellman M , Sennerby L. Direct implant loading in the edentulous maxilla using a bone density‐adapted surgical protocol and primary implant stability criteria for inclusion. Clin Implant Dent Relat Res. 2005;7(suppl 1):S60–S69. 16137089 10.1111/j.1708-8208.2005.tb00076.x

[31] Rodrigo D , Aracil L , Martin C , Sanz M . Diagnosis of implant stability and its impact on implant survival: a prospective case series study. Clin Oral Implants Res. 2010;21(3):255–261. 19958375 10.1111/j.1600-0501.2009.01820.x

[32] Sennerby L , Meredith N. Implant stability measurements using resonance frequency analysis: biological and biomechanical aspects and clinical implications. Periodontol 2000. 2008;47:51–66. 18412573 10.1111/j.1600-0757.2008.00267.x

[33] Oates TW , Valderrama P , Bischof M , et al. Enhanced implant stability with a chemically modified SLA surface: a randomized pilot study. Int J Oral Maxillofac Implants. 2007;22(5):755–760. 17974109

[34] Chambrone L , Shibli JA , Mercurio CE , Cardoso B , Preshaw PM . Efficacy of standard (SLA) and modified sandblasted and acid‐etched (SLActive) dental implants in promoting immediate and/or early occlusal loading protocols: a systematic review of prospective studies. Clin Oral Implants Res. 2015;26:359–370. 24814519 10.1111/clr.12347

